# Clinical outcomes of a short-term family-focused intervention for patients with atrial fibrillation–A randomised clinical trial

**DOI:** 10.1371/journal.pone.0282639

**Published:** 2023-03-16

**Authors:** Stine Rosenstrøm, Signe Stelling Risom, Thomas Kallemose, Ulrik Dixen, Jens Dahlgaard Hove, Anne Brødsgaard

**Affiliations:** 1 Department of Cardiology, Copenhagen University Hospital, Amager Hvidovre, Capital Region of Denmark; 2 Nursing and Health Care, Department of Public Health, University of Aarhus, Aarhus, Denmark; 3 Department of Cardiology, Herlev and Gentofte University Hospital, Hellerup, Denmark; 4 Institute of Nursing and Nutrition, University College, Copenhagen, Denmark; 5 Faculty of Health and Medical Sciences, Copenhagen University, Copenhagen, Denmark; 6 Department of Clinical Research, Copenhagen University Hospital, Amager Hvidovre, Capital Region of Denmark; 7 Dept of Clinical Medicine, University of Copenhagen, Copenhagen, Denmark; 8 Department of Paediatrics and Adolescent Medicine, Copenhagen University Hospital, Amager Hvidovre, Capital Region of Denmark; 9 Department of Obstetrics and Gynaecology, Copenhagen University Hospital, Amager Hvidovre, Capital Region of Denmark; Kurume University School of Medicine, JAPAN

## Abstract

**Aims:**

To evaluate a family-focused intervention for patients with atrial fibrillation (AF) in addition to conventional care and to establish its effect on health-related quality of life (HRQoL), anxiety, depression, AF symptoms, and family support.

**Background:**

AF is a widespread heart disease affecting the well-being of patients and their family members physically and psychologically. Supporting patients and their family members could potentially facilitate regaining family strength and improve HRQoL.

**Methods:**

Patients with newly diagnosed AF were randomised to standard care or additional family-focused intervention with change in global score of the Atrial Fibrillation Quality of Life Questionnaire (AFEQT) as primary outcome after six months’ follow-up. Secondary outcomes included the Hospital Anxiety and Depression Score, the European Heart Rhythm Association score, the Ice Expressive Family Functioning Questionnaire, and the Ice Family-Perceived Support Questionnaire (ICE-FPSQ).

**Results:**

Sixty-eight patients received standard care (n = 35) or family focused intervention (n = 33). The median change at the six-month follow-up on the global AFEQT score was 4.17 (-1.46–9.17) in the control group and 5.83 (-2.5–30) in the intervention group, yielding a median difference of -1.67 (p = 0.500). Change in ICE-FPSQ showed significant positive scores in favour of intervention (p < 0.001); other secondary outcome changes were non-significant.

**Conclusion:**

The family-focused intervention had a small positive but non-significant effect on HRQoL compared to standard care. To address the impact of AF on the patients and family members seems to improve anxiety and depression scores and perceived family support.

## Introduction

Atrial fibrillation (AF) is a widespread cardiac arrhythmia estimated to affect more than 20.9 million men and 12.6 million women worldwide [1]. The condition is among the leading causes of hospitalisation in patients with heart disease; and left untreated, AF may lead to stroke, heart failure, ischaemic heart disease and increased mortality [2, 3]. Treatment of AF is complex and requires a high degree of patient adherence and integrated care to prevent complications [4, 5]. Furthermore, AF substantially impacts the lives of patients and their family members [6–8], and health-related quality of life (HRQoL) is often negatively affected by anxiety and insecurity in both patients and their family members [9, 10]. Patients and family members both have a need for information and support to manage life with AF due to the nature of the disease. The arrhythmia can be associated with various symptoms including dyspnoea, chest pain and palpitations [11, 12], which affect patients and family members in various ways e.g. fear of what future will bring living with AF, fear of AF attacks and potentially fatal complications such as stroke [13, 14].

The European Society of Cardiology (ESC) guidelines recommend integrated AF care involving the family as a resource to ensure treatment compliance. Even so, the guidelines provide no instructions about how family involvement may be organised or facilitated in clinical practice [15]. Dalteg et al. found that couples in which one part had been diagnosed with AF often harboured core beliefs that AF was a life-threatening condition. The beliefs of patients and family members serve as a helpful cognitive map that nurses with advantage could encourage to share when meeting the family [8]. Focusing on the family as a unit rather than just the patient appears to improve problem-solving strategies and enhance rituals in the family that may improve physical and mental health [16–18] which potentially could impact HRQoL.

To our knowledge, no prior studies have examined the effect of a family-focused nursing intervention in patients with AF. Earlier intervention studies have focused on optimising treatment and adherence without focusing on the family [19–21]. Furthermore, studies examining the experiences of patients living with other chronic diseases and the effect of family-focused nursing have shown positive outcomes for patients and family members [22–24]. However, these experiences warrant further examination in different clinical settings. Due to the increasing incidence and prevalence of AF, the reduced HRQoL of the patients and the complexity of AF treatment, a robust set-up is needed to handle the disease, particularly after discharge and during the outpatient follow-up period. Therefore, this study aimed to evaluate a family-focused intervention for patients with AF in addition to conventional care and establish its effect on quality of life, anxiety and depression symptoms, and family support after six months of follow-up.

## Methods

### Trial design

The study was designed as a non-pharmacological, prospective, randomised trial with parallel groups in which patients were followed and assigned to either usual care or usual care plus a family-focused intervention. Intervention patients received a two-hour family group session and from one to three sessions of family-focused conversations with nurses. The trial followed the CONSORT (Consolidated Standards of Reporting Trials) guidelines for non-pharmacological interventions [25].

### Setting and data collection

Patients from two Danish university hospital out-patient clinics in the Capital Region of Denmark were screened consecutively for eligibility. Nurses from the clinics recruited patients and their family members when having a consultation with the patient in the outpatient clinic. The recruitment period was highly affected by the COVID-19 pandemic, which interrupted the study period due to the many lockdowns forcing the intervention to be paused repeatedly. The COVID-19 pandemic also challenged delivery of any interventions and care because patients and their families were allowed to visit the hospital only in acute cases from mid-March 2020 to mid-May 2020 and from December 2020 to January 2021. Furthermore, during the last six months of the intervention, patients and family members had to carry a face mask and comply with the distancing requirements of national COVID-19 guidelines. Because of COVID-19, many patients were afraid to visit the hospital, and several patients preferred telephone consultations or postponed their appointments. Therefore, the actual number of patients with AF and appointments in the out-patient clinic was lower than expected before COVID-19. Nevertheless, we found that patients and their families did not decline to participate in this study due to COVID-19; they were simply more careful when deciding when and how to receive care at the hospital.

The inclusion criterion was recently diagnosed AF (< 6 months) according to the ESC guidelines [15]. The exclusion criteria were left ventricular ejection fraction (LVEF) < 40%, inability to speak or understand Danish, co-morbidities associated with life expectancy < 1 year and mental disorder.

All patients were encouraged to identify a family member who would take part in the intervention. The concept of family was defined as *"family is who they say they are"* [26]. Therefore, the patient defined who he or she considered to be family. Thus, family could also be a neighbour or a close friend who participated in the intervention.

Study data were collected and managed using Research Electronic Data Capture (REDCap) electronic data capture tools hosted in the Capital Region of Denmark [27]. REDCap was also used for randomisation [28]. Allocation was concealed from investigators to minimise selection bias [29]. Patients were randomised in blocks of ten (five controls and five interventions) to balance the intervention with approximately five patients and family members entering the intervention every month. It was not possible to blind the intervention from patients, project nurses or investigators.

### Conventional treatment

The two recruiting outpatient clinics had similar standard care programs for AF patients. According to existing guidelines, the intervention group and the control group both received standard medical treatment for patients with AF [3].

The standard care and treatment program comprised at least one cardiologist consultation followed by at least one nurse consultation during which disease-specific information on AF was disseminated. The communication effort focused on informing the patient of the medication and treatment plans. All patients received information about anticoagulation treatment, other medical treatment of the arrhythmia and any relevant concomitant diseases, scheduled cardioversion and invasive catheter treatment, if needed. Patients were also offered attendance at the standard information program comprising a two-hour disease-specific group session for 8–10 AF patients during which an AF-specialised cardiology nurse informed about common elements of AF and treatments developed based on the ESC guidelines, and answered patients’ questions. The two-hour session focused on AF and its treatment.

### Family-focused intervention

The family-focused intervention was given in addition to the conventional treatment and care provided for patients with AF. The intervention was delivered based on the theoretical framework of family nursing, the Calgary Models and the Illness Belief Model [26, 30, 31] and knowledge from patients’ and family members’ needs when a patient is being diagnosed with AF [13, 14].

Patients and family members were offered the family-focused intervention according to their needs during a period of one to two months. Because the components in the intervention were tailored individually it was not possible to standardise the dose of the intervention. We did not exclude patients if they could not attend with a family member because the framework implicates a family perspective on how illness impacts the patients and the family, whether or not a family member is present [26, 30, 31].

The family-focused intervention comprised of a two-hour group education and 1–3 family nursing therapeutic conversations (FNTCs) delivered within two months. The intervention was considered delivered when patients and family members had participated in minimum one element of the intervention components (two-hour group session and or minimum one FNTCs).

Instead of a standard two-hour group education for patients with AF, intervention group families were first offered a two-hour educational family group session adapted to the theoretical framework of family nursing [26]. The size of the group was smaller (max five patients) than groups receiving the conventional group session and patients were invited to bring one family member each. Opposite the conventional group session, which was disease-specific and patient-centred, the educational family group sessions were family-centred and focused on the family as the unit of care [26]. Both family educational group sessions and the family FNTCs were facilitated by an AF-specialised nurse who had received intensive theoretical and practical training at the Linné University, Kalmar, Sweden in delivering the FNTCs based on the components showed in Table 1. Two nurses with > 15 years of expertise in cardiology followed the training program and subsequently conducted the family-focused intervention. As in the standard group sessions, themes for the family group sessions were identified according to the ESC guidelines (Table 1) [3], but clear changes were introduced in terms of how standard and intervention sessions involved family members. The standard and the intervention sessions differed. They both catered to participants´ information needs; however, the intervention group sessions were more focused on the family members´ needs which were ascertained from interviews with family members, patients´ and family members´ narration of their experience when facing an AF diagnosis and the impact it has on their everyday life [13, 14]. Furthermore, the group sessions in the intervention were consistently based on the questions and support needs of the participating patients and family members. The session was held in a large hospital meeting room. The nurse presented the contents of themes through a specifically developed Power-Point presentation shown on a projector at all group sessions to ensure that all patients and family members had the same basic information about AF. All sessions centred on the needs and questions of the patients and their family members. In the sessions, nurses employed their theoretical knowledge of family system nursing to facilitate the dialogue between the nurse and the families as well as dialogue within families, thereby increasing mutual responsiveness and enhancing the discussion of everyday life with AF [26]. During the session, family members were separated from patients, forming small group sessions discussing for 10–15 minutes of how they experienced their everyday life with a family member affected by AF. Patients, for their part, gathered in other small groups where they talked with each other about life with AF.

**Table 1:** The themes for the two-hour family educational group sessions.

○ *Families’ individual experiences with AF*
○ *What is AF*
○ *Symptoms of AF*
○ *Medication/anticoagulation*
○ *Catheter ablation*
○ *Cardioversions*
○ *Lifestyle: diet, smoking, alcohol, and exercise*
○ *Psychological reactions*

AF: atrial fibrillation

Each family was invited to participate in up to one to three 60-minute FNTC sessions depending on their needs. The FNTCs were based on the Calgary Family Assessment Models (CFAM) and the Calgary Family Intervention Models (CFIM) in which the whole family is considered the unit of assessment and intervention [32]. Therefore, focus was not only on the patient but also on the interaction and reciprocity between family members, the patient and the nurse [26]. During the FNTCs, nurses employed circular questions, e.g., difference questions, behavioural-effect questions, and hypothetical or future-orientated questions like “How do you look at your life with AF?”, “Do you feel the same way as your family member?”, “What is most important for you and your family living with AF?” and “What is important to change in your everyday life?”. Therapeutic questions were used to facilitate a change in the cognitive, affective or behavioural domains of family functioning [26]. Furthermore, all families were asked the “One Question-Question", designed to elicit the patients’ and the family members’ most pressing needs or concerns [33]. The nurses also used a genogram, which is a drawing of the family constellation prepared in collaboration with the patient and the patient’s family member (Table 2). Moreover, the FNTCs focused on the patients’ and family members’ mutual and possibly diverse beliefs about AF and everyday life. The nurses’ role was to share knowledge about AF and to facilitate, inspire, motivate and strengthen patients’ and family members’ ability to manage life with AF. Furthermore, if the patient attended a session unaccompanied, the FNTCs focused on how AF influenced the family and social relationship. The contents of the FNTCs are shown in Table 2.

**Table 2:** Contents of FNTCs based on: *A guide to family assessment and intervention* [26].

Contents in the Family Nursing Therapeutic Conversations (FNTCs) with patients with atrial fibrillation (AF) and family members (FMs)
❖ Asking patients and FMs about their expectations regarding the family conversation and which issues and problems they consider most important to discuss concerning AF and their everyday life. ❖ Individual information, supervision and education following evidence-based clinical AF guidelines. ❖ Drawing a genogram reflecting a model of the family structure and social relationships. ❖ Encouraging patients and FMs to tell their family illness narrative and reflect upon their AF diagnosis beliefs. ❖ Identification of goals for the families’ future living with AF. ❖ Conversations about the impact of AF on the family unit and the individual FMs’ daily lives. ❖ Circular questions and the One-Question-Question focusing on strengths and resources within the family. ❖ Evaluation of the intervention based on conversation, observation, goal setting and perception of the family.

### Adherence to the intervention

All FNTCs were digitally recorded, allowing the research team to evaluate if the family-focused intervention had been delivered as intended. In practice, this was done by the first author who revised 19 randomly selected recordings to establish if the components in Table 2 had been included in the FNTCs. FNTCs were delivered in accordance with the family’s needs which were determined jointly by the nurse, the patient and the patient’s family member. Furthermore, the adherence to the intervention was registered in REDCap by first author documenting the attendance to the two-hour group session and the number of FNTC´s patients and family members accepted.

### Outcomes

The patients completed a set of self-reported questionnaires, tapping answers directly into the data management program REDCap using an Ipad before receiving the intervention and at a six-month follow-up visit. The baseline and follow-up visits were conducted in the out-patient clinic and facilitated by the first author who was available if patients and family members had any questions or experienced technical problems with the Ipad.

The primary outcome was defined as a clinically relevant change of 11 points on the global score measured on the Atrial Fibrillation Effect on Quality of Life Questionnaire (AFEQT). Secondary outcomes were changes in the Hospital Anxiety Depression Scale (HADS), the European Heart Rhythm Association Scale (EHRA), the Ice Expressive Family Functioning (ICE-EFFQ), and the Ice Family Perceived Support (ICE-FPSQ). Additionally, we registered adherence to the intervention by counting how many family sessions the patients and their families attended.

### Measurements

The AFEQT questionnaire is a self-administered disease-specific licensed and validated questionnaire sensitive to clinical changes in patients with AF [34–36]. The questionnaire has 20-items on a 7-point likert scale that ranges from the most severe limitation/symptom (7 points) to no limitation/symptom (1 point). Patients are asked to indicate how AF impacts their health within the previous four weeks in the questionnaire and impact of HRQoL is assessed by four domains: symptoms (4 items), daily activities (8 items), treatment concerns (6 items) and treatment satisfaction (2 items)(St Jude Medical, St Paul, MN, http://afeqt.org/) [34]. The questionnaire is not validated in Danish but translated to Danish by St.Medical. An overall global AFEQT score is calculated by using the first three domains and transformed to a score between 0–100. The overall global AFEQT score was calculated based on the following formula [34]

$$[100-\frac{\begin{array}{c} (sum\;of\;severity\;for\;all\;questions\;answered\;\\ -number\;of\;questions\;answered)\;x\;100 \end{array}}{\left(total\;number\;of\;questions\;answered\;x\;6\right)}].$$

A high score, close to 100, indicates a high (good) HRQoL and a low score close to 0 a low HRQoL. Treatment satisfaction is not calculated in the overall global AFEQT score in the survey. Furthermore, for each domain (Symptoms, Daily activities, Treatment concerns and Treatment satisfaction) there is a corresponding subscale score, which also ranges from 0–100 (0 = worst and100 = best).

The original questionnaire was generated with input from patients and was found to be feasible, reliable, valid and responsive to treatment [34]. Furthermore, the AFEQT was shown to be internally reliable owing to high Cronbach alpha coefficients (0.88) [34], and test-retest reliability was stable for the following domains: Global score, Daily Activities, Treatment Satisfaction, and Treatment Concern domain scores [34].

The HADS is licensed and validated in Danish to measure the level of depression and anxiety [34, 35]. It is a 14-item questionnaire employing a four-point Likert scale ranging from 0 to 3. Items enquire about how the patient has felt the past seven days. Half of the questions measure the level of depression; the other half, anxiety levels. Scores are calculated for anxiety and depression separately (score range 0 to 21). A score between 11 and 21 indicates an abnormal level of anxiety or depression. A score from 8–10 indicates borderline levels of anxiety or depression; a score from 0 to 7, normal levels [37]. The Danish validation of HADS showed internal consistency with a Cronbach´s alpha of 0.87 regarding HADS-A and 0.82 for HADS-D [38]. It is estimated that a 1.7 point change on HADS can be defined as relevant clinical difference [39].

The EHRA score is a well-known and validated practical semi-quantitative tool to measure relevant change in patients’ AF-related symptoms and perceptions of general state of health from the perspective of the patient [40] and it is translated to Danish. It measures the impact of AF episodes and symptoms on daily activities using a five-scale classification, ranging from no symptoms to disabling symptoms [3].

The ICE-EFFQ and the ICE-FPSQ are questionnaires that measure family functioning in families experiencing acute or chronic illness and patients and family members perceived support when offered family-focused in the healthcare system [41]. The validity and reliability were tested in a Danish version and found usable in the Danish context with a Crohnbach alpha on 0.93 in the ICE-EFFQ and 0.94 in the ICE-FPSQ [42]. ICE-EFFQ scores range from 17 points to a maximum score of 85 points measured on four domains of family function (Expressive emotions (4-items), Collaboration/Problem solving (5-items), Communication (4-items) and Behaviour (4-items)); ICE-FPSQ scores from a minimum of 14 points to a maximum of 70 points on two domains of perceived support (Cognitive (5-items) support and Emotional support (9-items)). Both questionnaires are answered on a five-point Likert type scale ranging from 1 (almost never) to 5 (all the time). High scores indicate better family-function (close to 84) [41] and better perceived support (closer to 70) [43]. However, there is no consensus in literature of what constitutes a relevant change [42]

### Sample size

A sample size of 50 patients in two arms (a total of 100 patients) was deemed sufficient for detection of an 11-point difference on the AFEQT with an expected standard deviation of 20, a power of 80% and a significance level of 0.05. Calculation was based on a two-sided independent two-sample t-test. When measuring HRQoL from a patient perspective an 6–19 point increase has been deemed to reflect a meaningful improvement in HRQoL measured by the AFEQT [44]. Therefore, we chose 11 point being a middle value of this interval.

### Statistical analysis

Continuous variables are presented as mean with standard deviation (SD) or median with interquartile range (IQR) depending on distribution of the variable. Categorical variables are presented as counts and percentiles. The within group change was calculated as follow-up minus baseline and presented as median. The change difference was calculated as control minus intervention. Changes from baseline to follow-up between intervention and standard care group were tested using the Wilcoxon rank-sum test because the normality assumption of the distribution could not be satisfied. The normality assumption was evaluated using QQ plots and histograms.

Given that the intervention is offered to the patients depending on their needs in order to mirror clinical practice, the analysis is considered intention to treat (ITT).

To account for possible differences in baseline values between groups (see the Results section for more details) adjusted quantile regression models for difference in median change were fitted as a sensitivity analysis. The models included group and the baseline value as parameters, and p-values were calculated from rank test based on the Wilcoxon score. 95% Confidence intervals (CI) for the change in median difference were also estimated from quantile regression models both adjusted and unadjusted. Be aware that CI for the difference in medians are based on the median comparison and the rank test considers the entire distribution, since the median is only used at a “surrogate” measure for the ranks some discrepancies between the rank test p-value and median CI may occur. Interpretation of the CI’s should therefore only be interpreted with regards to the precision/certainty of the estimated difference in the median change.

Missing data were considered missing at random. No further imputation of missing values was made. Statistical significance was defined as a p-value below 0.05. All analyses were conducted using R version 3.6.1 [45]. Given that no patients were excluded due to failure to complete the intervention, results are interpreted as having been made on an intention-to-treat basis.

### Ethical considerations

All patients and family members received written and oral information about the trial and gave written consent before participating [46]. Patients and families were informed that all their data would be securely stored on an encrypted drive, and that their identities would be protected when reporting the findings. Patients and family members were informed that they could withdraw from the study at any time. Testing this intervention, we ensured that the families experienced a safe environment throughout the intervention and that interaction with the families was always conducted with respect and dignity. In all family contact, we were conscious of the need to maintain the delicate balance between the needs of the patients and the family members by using non-confrontational communication techniques [47]. Furthermore, we conducted the trial according to the fundamental ethical principles to ensure no harm, either intentional or unintentional. The trial was approved by the Danish Protection Agency (VD-2019-42) and the Scientific Ethical Committee of the Capital Region of Denmark (id:19007769), and it was registered with ClinicalTrials.Gov (NCT04165421). Furthermore, we conducted the trial in accordance with the Helsinki Declaration and the International Committee of Medical Journal Editors (IMJE) Recommendations for the Protection of Research Participants [48, 49].

## Results

**Fig 1:**
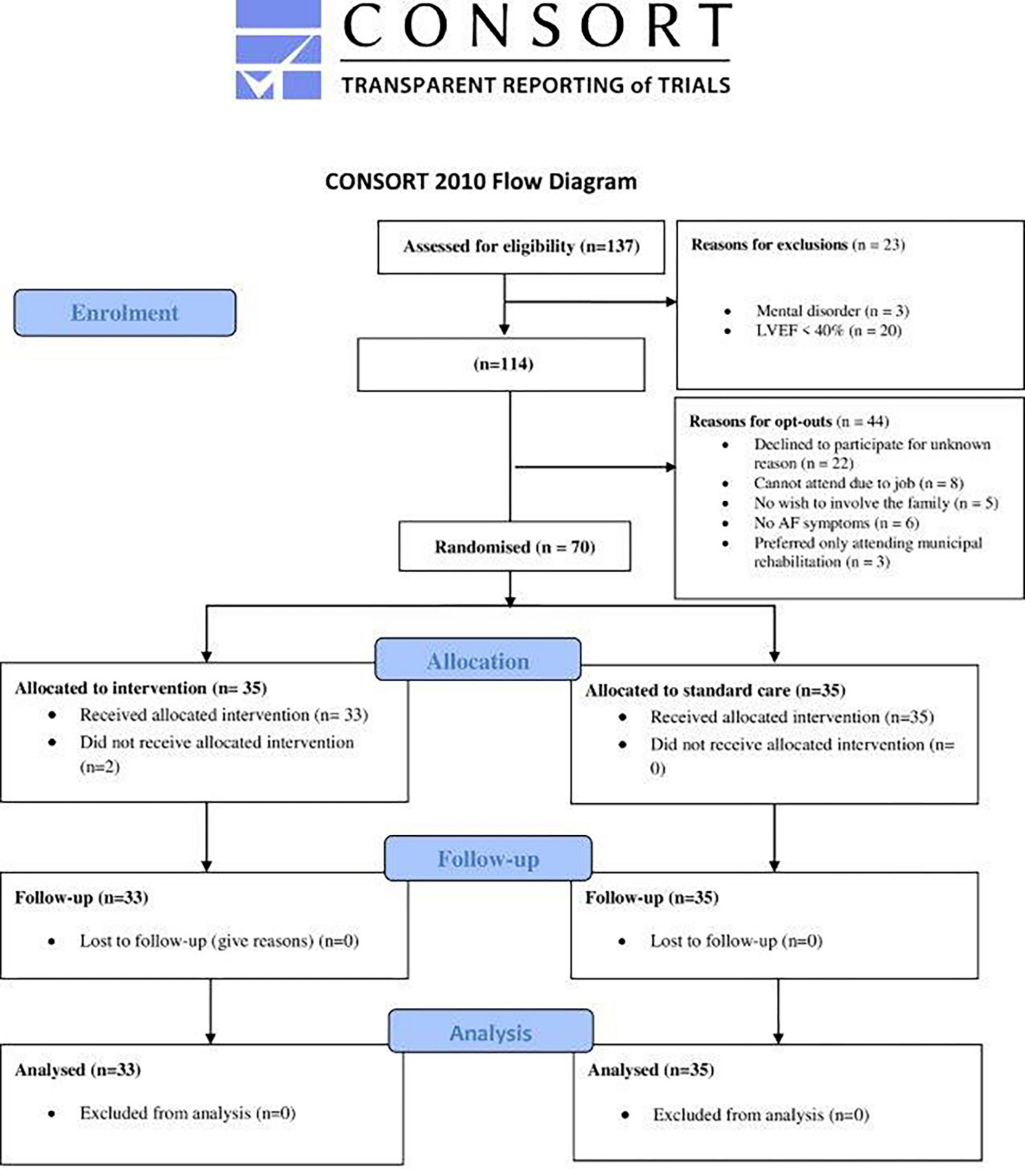
Consolidated standards of reporting trials (Consort) flowchart.

The original plan was to recruit ten patients with a family member per month during a 12-month period from November 2019 (first patient recruited 28^th^ of November) 2019 to January 2021. However, due to COVID-19 and associated lockdown periods, the recruitment period was extended to March 2021. In that period, we approach 137 patients with AF of whom 108 were eligible and 70 (control group n = 35; intervention group n = 35) accepted to participate. Two patients and their families withdrew their consent after inclusion and before starting the intervention; one patient had knee surgery and the other patient had lost a close family member. The last patient was had follow-up in September 2021. Data on enrolment of families, allocation and follow-up are summarised in the CONSORT flow diagram (Fig 1).

The mean age of the patients was 68 years in both groups. The intervention group (n = 33) counted 22 male patients and 11 female patients; the control group (n = 35), 22 male patients and 13 female patients. Patients’ AF symptoms varied considerably; from almost no symptoms to symptoms disabling their everyday life. Twenty-two of the enrolled intervention patients were accompanied by their spouses; four patients, by adult son or daughter; three patients, by a friend; and four patients attended alone because they had no family member who could accompany them. The groups were considered similar at baseline as no clinical difference was observed between the groups for the baseline variables, all patient characteristic at baseline are presented in Table 3. Data on adherence to the two-hour group sessions and the FNTCs are shown in Table 4. Regarding the 33 patients attending the family-focused intervention, the intervention was conducted with the patient and a family member 85% of the time. The rate of missing values in the questionnaires was low; three answers were missing from the AFEQT questionnaire in the intervention group at baseline, two answers were missing from the control group at follow-up and two answers were missing from the intervention follow-up. No deaths or serious adverse advents were registered during the study.

**Table 3:** Baseline characteristics of randomised patients.

Baseline characteristics of randomised patients	Intervention group N = 33	Control group N = 35
**Age**	68 (64.0: 74.0)	68 (63.0: 72.5)
Male	22 (0.67)	22 (0.63)
Female	11 (0.33)	13 (0.37)
**Patient reported AF**		
Persistent	3 (0.09)	10 (0.29)
Paroxysmal	13 (0.39)	13 (0.37)
Permanent	6 (0.18)	3 (0.09)
Not known by patient	11 (0.33)	9 (0.26)
**Time since AF diagnosis**		
< 1 month	10 (0.30)	7 (0.20)
> 1 - < 3 months	12 (0.36)	14 (0.40)
> 3 - < 5 months	6 (0.18)	11 (0.31)
> 5 -<6 months	5 (0.15)	3 (0.09)
**AF symptoms**		
Palpitations	21 (0.64)	19 (0.54)
Angina	12 (0.36)	8 (0.23)
Dyspnea	22 (0.67)	21 (0.60)
Fatigue	18 (0.55)	15 (0.43)
Syncope	1 (0.03)	0 (0.00)
No symptoms	4 (0.12)	5 (0.14)
**BMI**	28.1 (24.4:31.9)	26.9 (24.2:32.1)
**Current smoker**	2 (0.06)	2 (0.06)
**Prior treatment**		
Electrical cardioversion	5 (0.15)	13 (0.37)
Catheter ablation	1 (0.03)	2 (0.06
**Anticoagulation**		
NOAC	30 (0.91)	34 (0.97)
Warfarin	2 (0.06)	0 (0.00)
None	1 (0.03)	1 (0.03)
**Antiarrhythmic treatment**		
Beta-blockade	22 (0.67)	22 (0.63)
Calciumblockade	0 (0.00)	4 (0.11)
Digoxin	5 (0.15)	4 (0.11)
**Other antiarrhythmic drugs	2 (0.06)	2 (0.06)
**Other treatment**		
Diuretics	5 (0.15)	13 (0.37)
Antidepressants	1 (0.03)	2 (0.06)
**Comorbidity**		
Other cardiovascular disease	9 (0.28)	16 (0.46)
Diabetes	3 (0.09)	4 (0.11)
Musculoskeletal symptoms	11 (0.33)	7 (0.20)
Lung disease	5 (0.15)	3 (0.09)
Thyroid disease	3 (0.09)	1 (0.03)
**Matrimonial status, n (%)**		
Married	23 (0.72)	26 (0.76)
Single	4 (0.12)	3 (0.09)
Cohabiting	2 (0.06)	2 (0.06)
Divorced	3 (0.09)	1 (0.03)
Widower	0 (0.00)	2 (0.06)
**Education, n (%)**		
10 years or less	9 (0.27)	4 (0.12)
High school	3 (0,09)	1 (0.03)
Vocational	8 (0.24)	11 (0.31)
Higher education	12 (0.36)	19 (0.54)
**Employment status, n (%)**		
Work	10 (0.30)	13 (0.39)
Unemployed	0 (0.00)	2 (0.06)
Retired	23 (0.70)	18 (0.55)

*Baseline data are self-reported **Other antiarrhythmic drugs, e.g,. amiodarone.

*AF: atrial fibrillation. BMI = Body Mass Index. *NOAC = Novel oral anticoagulation.

All categorical variables are presented as frequencies with percentages.

All continuous variables are presented as median with interquartile range.

**Table 4:** Patient adherence to two- educational group session and number of FNTC´s.

*Adherence to the elements in the intervention	N (%)
Two-hour family educational group session and 1 FNTC	16 (50)
Two-hour family educational group session and 2 FNTC’s	12 (36)
Two-hour family educational group session and 3 FNTC’s	2 (6)
Two-hour family educational group session, and > 3 FNTC’s	1 (3)
Two-hour family group educational group session none FNTC	1 (3)
No two-hour family group educational group session and 1 FNTC	1 (3)
Total	33 (100)

*FNTC: family nursing therapeutic conversation

*Adherence was calculated as how many elements each patient attended during the intervention period

### Intervention effect on the primary outcome and secondary outcomes

At baseline, a difference was observed between the groups’ self-reported HRQoL measured on the global AFEQT score with a median score of 76.67 (61.67–80.83) in the control group versus 63.75 (47.50–78.54) in the intervention group. The median change at the six-month follow-up on the Global AFEQT score was 4.17 (-1.46–9.17) in the control group compared with 5.83 (-2.5–30) in the intervention group, yielding a median difference of -1.67 (CI = -9.77–2.27, p = 0.500). We did not find any significant changes within the subscales; however, we did observe a large variation within the intervention group compared to the control group based on interquartile ranges and CI’s (symptoms, daily activities, treatment concerns. See Table 5 for detailed result values.

**Table 5:** Primary outcomes on AFEQT.

Variable	Control group baseline N = 35	Intervention group baseline N = 33	Control group follow-up N = 35	Intervention group follow-up N = 33	Control group change (follow-up—baseline)	Intervention group Change (follow-up—baseline)	Change Difference (control—intervention)	p-value*
**AFEQT**								
AFEQT global score	76.67 (61.67–80.83)	63.75 (47.50–78.54)	80.42 (68.75–87.08)	79.58 (68.12–85.83)	4.17 (-1.46–9.17)	5.83 (-2.50–30.00)	-1,67 (-9.77–2.27)	0.500
Symptoms	83.33 (70.83–91.67)	70.83 (58.33–91.67)	91.67 (75.00–100.00)	83.33 (75.00–95.83)	6.25 (-4.17–12.50)	4.17 (0.00–20.83)	2.08 (-9.95–14.12)	0.600
Daily activities	82.61 (59.78–89.13)	59.78 (37.50–89.13)	84.78 (61.96–98.91)	84.78 (63.04–93.48)	6.52 (-6.52–11.96)	7.61 (-0.54–33.15)	-1.09 (-11.76–5.24)	0.300
Treatment concern	77.78 (56.94–87.50)	68.06 (49.31–83.33)	88.89 (72.22–95.83)	91.67 (80.56–100.00)	8.33 (0.00–16.67)	16.67 (5.56–34.72)	-8.33 (-16.34–5.23)	0.100
Treatment satisfaction	33.33 (12.50–50.00)	41.67 (33.33–50.00)	16.67 (0.00–29.17)	25.00 (16.67–33.33)	-8.33 (-29.17–4.17)	-16.67 (-33.33–0.00)	8.33 (-24.48–32.81)	0.300

All estimates except change difference are presented as median with interquartile range, change difference presented as difference in median change with 95% confidence interval. AFEQT: atrial fibrillation Quality of life.

*p values are based on Wilcoxon sum-rank test comparing control and intervention change

Outcomes on anxiety and depression showed decreased levels at follow-up for both groups, but the intervention group recorded a larger, but non-significant, decline in their scores (HADS-A: median change -3: -5.00 - -1.00) and (HADS-D: median change -1: -5.00–0.00). EHRA scores also decreased in both groups from 3 to 2 during the six-month follow-up. The ICE-EFFEQ showed positive, but non-significant, changes in all domains, except for communication with a difference in median change of -1.00 (CI = -2.94 –-1.00, p = 0.027). The ICE-FPSQ-total showed significant positive scores in all domains in favour of the intervention (all p < 0.001). See Table 6 for full values.

**Table 6:** Secondary outcomes on the HADS, EHRA, ICE-EFFQ and ICE-FPSQ.

Variable	Control baseline, N = 35	Intervention baseline, N = 33	Control follow-up, N = 35	Intervention follow-up, N = 33	Control change	Intervention change	Change difference	p-value
**HADS**								
Anxiety	3 (1.00–6.00)	7 (4.00–9.00)	1 (0.00–4.00)	3 (1.00–5.00)	-1 (-5.00–1.50)	-3 (-5.00 - -1.00)	2 (0.06–5.94)	0.100
Depression	1 (0.00–4.00)	4 (2.00–6.00)	0 (0.00–2.50)	2 (1.00–4.00)	0 (-2.00–1.00)	-1 (-5.00–0.00)	1 (0.062–1.00)	0.100
**EHRA score**	3 (2.00–3.00)	3 (2.00–4.00)	2 (1.00–3.00)	2 (1.00–3.00)	0 (-1.00–0.00)	-1 (-2.00–0.00)	1 (-1.94–1.00)	0.600
**ICE-EFFQ**								
Expressive emotions	18.00 (16.00–18.00)	16.00 (14.00–18.00)	18.00 (17.00–20.00)	18.00 (16.00–19.00)	1.00 (0.00–2.00)	1.00 (-1.00–3.00	0 (-1.94–3.94)	0.916
Collaboration/problem solving	21.00 (20.00–22.00)	20.00 (19.00–22.00)	23.00 (21.00–24.50)	22.00 (20.00–25.00)	1.00 (-1.00–3.00)	2.00 (-1.00–5.00)	-1.00 (-1.94–2.94)	0.517
Communication	16.00 (14.00–18.00)	15.00 (12.00–16.00)	16.00 (14.00–19.00)	16.00 (15.00–20.00	1.00 (-0.50–2.00)	2.00 (0.00–4.00)	-1.00 (-2.94 –-1.00)	0.027
Behaviour	17.00 (15.00–18.00)	16.00 (15.00–17.00)	17.00 (16.00–20.00)	18.00 (16.00–20.00)	0.00 (-1.00–2.50)	2.00 (0.00–4.00)	-2.00 (-2.00–2.94)	0.111
**Total ICE-EFFQ**	72.00 (66.00–75.00)	67.00 (63.00–74.00)	76.00 (68.50–79.50	76.00 (66.00–80.00)	4.00 (-1.00–8.00)	6.00 (2.00–13.00)	-2.00 (-6.88–2.88)	0.110
**ICE-FPSQ**								
Cognitive support	14.00 (11.00–18.50	15.00 (11.00–21.00)	15.00 (11.00–20.00)	24.00 (22.00–25.00)	2.00 (-2.50–5.00)	7.00 (2.00–12.00)	-5.0 (-9.94 –-1.06)	<0.001
Emotional support	9.00 (9.00–17.00)	11.00 (9.00–21.00)	10.00 (9.00–16.00)	39.00 (32.00–41.00)	0.00 (-2.00–3.50)	22.00 (7.00–29.00)	-22.00 (-23.94 –-18.06)	<0.001
**Total ICE-FPSQ**	24.00 (20.50–34.00)	26.00 (21.00–43.00)	28.00 (21.50–35.50)	64.00 (54.00–66.00)	5.00 (-3.50–8.00)	24.00 (15.00–40.00)	-19.00 (-34.94 –-16.12)	<0.001

All estimates except change difference are presented as median with interquartile range, change difference presented as difference in median change with 95% confidence interval.

HADS: Hospital Anxiety and Depression Scale. EHRA score: European Heart Rhythm Association score. ICE-EFFQ: Ice Expressive Family Functioning. ICE-FPSQ: Ice Family Perceived Support questionnaire.

Sensitivity analysis adjusting for baseline values showed a larger estimated difference on Global AFEQT score -4.21 (CI = -9.33–9.01, p = 0.910). The only estimate leading to a different interpretation of the result was for ICE-EFFEQ communication having a non-significant difference in median change -0.86 (CI = -1.80 –-0.021, p = 0.149). All estimates and p-values for adjusted analysis are presented in S1 File.

## Discussion

No statistically significant improvement from the intervention was found for the six months change on Global AFEQT score, compared to standard care. Additionally, confidence intervals for the estimates were wide, giving a large uncertainty and making interpretation about the size of the difference hard. Adjustments for baseline values did result in a larger estimated difference, however the uncertainty for this estimate was even larger than the uncertainty for the unadjusted estimate and thereby not making interpretation of the result any easier. Because of this large uncertainty a possible relevant effect may be present, but it is not possible to answer from the data in this study.

For secondary outcomes only ICE-FPSQ Cognitive, emotional and total score showed a significant difference and reasonable thin confidence intervals, indicating a possible difference might be present. This would have to be confirmed in another study. However, there is no consensus in literature og what constitutes a relevant change [42].

Supportive of the possible future use of family-focused nursing as an integrated part of AF care and treatment, this intervention was shown to be feasible in an out-patient clinical setting even during the COVID-19 pandemic. Notably, approximately half of the eligible and approached patients and their families accepted participation in the study.

According to Dorian et al., 18.9 increase in the global AFEQT score is considered a moderate improvement and 27.8 a large improvement [44]. In our study, most patients in both groups reported at least moderate improvements in HRQoL at follow-up. As the difference between the groups was not statistically significant, we cannot conclude that the intervention caused the effect. However, the chosen significance level, an 11-score point increase, may have been a too ambitious goal threshold given the high level of our standard care and treatment offer. Furthermore, changes in AFEQT +/- 5 points have been estimated to be clinically relevant, though these estimates are from another AF study population and based on an anchorbased method where patients’ responses on the AFEQT are compared to the EHRA score from a clinician’s perspective [35].

Results similar results to ours were observed in a randomised controlled study investigating the effect of nursing interventions targeting patients with heart failure and their family members; due to lack of power, this study detected no significant effect on HRQOL, anxiety or depression [22]. As in our study, the secondary outcomes on the ICE-FPSQ scale showed positive changes in the intervention group [22].

Even though none of the patient in the control or intervention group had high scores on the HADS at baseline, we observed a decrease in anxiety and depression scores in both groups though the intervention group experienced more positive (50%) changes reflected in the AFEQT scores, which could be a clinical relevant change on HADS [39]. Other studies have shown that the burden of AF is associated with higher rates of anxiety and depression, and psychological stress, which may increase mortality [50]. Therefore, patients with AF should be assessed for depression and anxiety [51]. Our study show that it is feasible to measure both the AFEQT and the HADS and that over time patients had higher HRQoL scores and lower anxiety and depression scale scores when treated and receiving care.

Whether our results should pave the way for a more systematic use of family-focused intervention may be discussed with a focus on the evaluation method and the study context. The present study can be categorised as a complex intervention within the context of the healthcare system where multiple factors may influence outcomes, actions and behaviour of patients, families and health professionals [52]. Therefore, evaluating the family-focused intervention, one should be aware of factors within the context that may affect the intervention and its outcomes [53]. In this study factors such as e.g. COVID-19 resulted in several disruptions of the scheduled appointments for both the intervention group and the control group. Furthermore, the intervention was highly depending on the project-nurses’ availability and engagement.

Other interventions targeting HRQoL in AF patients have used AFEQT to measure quality of life [54]. Patients with AF undergoing radiofrequency ablation experienced a clinically relevant improvement on the AFEQT > five points compared with patients undergoing antiarrhythmic drug treatment, and they had baseline values similar to those found in our study [55]. Radiofrequency ablation increased patients’ HRQoL when measured in a homogeneous group highly affected by AF symptoms. However, several elements including the complexity of the context and the intervention must be considered when our AF intervention is compared with other interventions using the same primary outcome. Our study included patients presenting a wide range of symptoms, from almost no symptoms to multiple arrhythmia symptoms. We also included patients with several other co-morbidities than AF, which could have worsened the patients´ general life situation. Furthermore, the intervention focused on supporting cognitive, emotional and social capacities in patients and family members living with AF. Therefore, in this heterogeneous group, it may be difficult to detect large differences in HRQoL due to the presence of other concurrent chronic conditions. Notwithstanding, the present intervention focused on psychological well-being through education, support and guidance rather than doing a simpler less-or-more AF symptoms comparison.

Another relevant aspect to consider is that the family-focused intervention may potentially have affected other patient-reported outcomes than those we chose, e.g., emotional wellbeing and self-efficacy, which is the individual’s belief in his or her capacity to manage specific tasks and feeling self-confident [56].

Nevertheless, we provided reflective space for expressing illness beliefs and insecurity among patients and their family members. The present study focused on patient-related outcomes. However, it could also have been designed to measure family member outcomes as well. Petursdottir et al. showed that a family therapeutic intervention yielded increased perceived support, decreased stress and reduced caregiving demands among the family members [57]. Furthermore, the dose of intervention (group session FNTCs), the combination of intervention elements, the timing of the intervention and evaluating the right outcomes require further work to estimate the effects of family-focused nursing in complex social and clinical settings.

### Strengths and limitations

The methodology of the study using an intervention group vs. a control group in a RCT design is a strength in order to avoid systematic errors and the intervention and the control group were not clinically different from each other in the descriptive baseline characteristics, possible differences were present for outcomes measures at baseline, however the sensitivity analysis showed this only impacted the result of a single secondary outcome. Due to COVID-19, the inclusion period was disrupted, and the patient inclusion rate was lower than expected. Thus, the study ended up being underpowered (control group n = 35; intervention group n = 33) for assessing the effect of the family-focused intervention on the AFEQT score. Furthermore the 11 point change on the AFEQT used in the sample size calculation could have been reduced to 5 [35] such that the sample size calculation could detect all relevant changes. In addition, it is a limitation that we have used the AFEQT questionnaire originally developed and validated for up to three months of follow-up whereas here, it was used in a six-months follow-up design. Moreover, the longer follow-up period in this study could allow other therapeutic interventions to affect the HRQoL measured from the AFEQT. Additionally, there is a gap of knowledge in the literature regarding a clinically meaningful change in AFEQT, and there is variation in the literature depending on which anchor-based assessment has been used, which can result in different values and interpretation of the results [35, 44]. possible dropouts were not considered during sample size calculation but should have been included, which would have resulted in a larger sample size being required. These limitations on the sample size increase the risk of TYPE II errors. They also account for the wide confidential intervals.

The generalisability of the study findings is limited due to the COVID-19 pandemic, resulting in patient receiving. intervention/standard care as early as intended, thereby impacting the effect of the intervention. Additionally, the pandemic probably affected the mental and physical well-being of the participating patients and their families. However, we expect that the negative effects of COVID- 19 have equally affected the intervention and control group. Because the intervention potentially allowed participants to only undergo one session in total there is a possibility that different participants received different amount of the intervention, which could introduce bias in the results. Furthermore, the intervention was delivered in the course of a year. The nurses might have improved their family-focused nursing skills during the trial, reducing the effect at the beginning of the trial, checked during the intervention this was not revealed as a problem. It was not possible to blind clinicians due to the nature of the intervention. Therefore, the open-label design of the study may have biased the results in favour of the intervention because the follow-up visit was conducted by the first author who also recruited patients and their family members. It was a strength in this study that all FNTCs were recorded digitally and 19 recordings were validated by the first author to confirm that the components of the intervention were delivered as intended and that the FNTCs were individualised and met families’ needs. An additional strength is that nurses delivering the family-focused intervention received the same skills training within a theoretical and practical, certified post-graduate training programme (Linné University, Sweden) with experienced teachers and examinations to conclude the course. Furthermore, both nurses were actively involved in the planning and development of the intervention, which enhanced ownership to the intervention. Given the complex nature of this trial and the mentioned limitations, our findings should be considered preliminary and should be interpreted with caution. The fact that patients in the intervention group in this sample had lower scores at baseline could indicate a positive effect of the intervention even though the study was underpowered. Therefore, results warrant examination in a higher-powered study. Furthermore, it could be relevant to broaden a future study to also measure the effect on the family members as well as the patient, which also could support knowledge of the effect of a family-focused approach.

## Conclusion

Family-focused intervention did not show an improvement on the global AFEQT score compared to traditional treatment. However, given the large uncertainty of the estimates it is possible that relevant effects could be present, because of this no conclusive answer about this association can be made based on this study.

## Supporting information

S1 Checklist CONSORT 2010 checklist of information to include when reporting a randomised trial*. (DOC)

S1 File (DOCX)

S2 File (PDF)

## References

[1] Chugh SS, Havmoeller R, Narayanan K, Singh D, Rienstra M, Benjamin EJ, et al. Worldwide Epidemiology of Atrial Fibrillation: A Global Burden of Disease 2010 Study. Circulation. 2014 Feb 25;129(8):837–47. doi: 10.1161/CIRCULATIONAHA.113.005119 24345399 PMC4151302

[2] Ringborg A, Nieuwlaat R, Lindgren P, Jönsson B, Fidan D, Maggioni AP, et al. Costs of atrial fibrillation in five European countries: results from the Euro Heart Survey on atrial fibrillation. Eur Eur Pacing Arrhythm Card Electrophysiol J Work Groups Card Pacing Arrhythm Card Cell Electrophysiol Eur Soc Cardiol. 2008 Apr;10(4):403–11. doi: 10.1093/europace/eun048 18326853

[3] Hindricks G, Potpara T, Dagres N, Arbelo E, Bax JJ, Blomström-Lundqvist C, et al. 2020 ESC Guidelines for the diagnosis and management of atrial fibrillation developed in collaboration with the European Association of Cardio-Thoracic Surgery (EACTS). Eur Heart J. 2020 Aug 29;ehaa612.

[4] Brandes A, Department of Cardiology, Cardiology Research Unit, Odense University Hospital, University of Southern Denmark, Odense, Denmark, Smit MD, Thoraxcentre, University of Groningen, University Medical Centre, Groningen, The Netherlands, Nguyen BO, Thoraxcentre, University of Groningen, University Medical Centre, Groningen, The Netherlands, et al. Risk Factor Management in Atrial Fibrillation. Arrhythmia Electrophysiol Rev. 2018;7(2):118. doi: 10.15420/aer.2018.18.2 29967684 PMC6020195

[5] Kirchhof P. The future of atrial fibrillation management: integrated care and stratified therapy. Lancet Lond Engl. 2017 Oct 21;390(10105):1873–87. doi: 10.1016/S0140-6736(17)31072-3 28460828

[6] McCabe PJ, Schumacher K, Barnason SA. Living with atrial fibrillation: a qualitative study. J Cardiovasc Nurs. 2011 Aug;26(4):336–44. doi: 10.1097/JCN.0b013e31820019b9 21263348

[7] Altiok M, Yilmaz M, Rencüsoğullari I. Living with Atrial Fibrillation: An Analysis of Patients’ Perspectives. Asian Nurs Res. 2015 Dec;9(4):305–11. doi: 10.1016/j.anr.2015.10.001 26724239

[8] Dalteg T, Sandberg J, Malm D, Sandgren A, Benzein E. The heart is a representation of life: an exploration of illness beliefs in couples living with atrial fibrillation. J Clin Nurs. 2017 Nov;26(21–22):3699–709. doi: 10.1111/jocn.13742 28122413

[9] Dalteg T, Benzein E, Sandgren A, Malm D, Årestedt K. Associations of Emotional Distress and Perceived Health in Persons With Atrial Fibrillation and Their Partners Using the Actor-Partner Interdependence Model. J Fam Nurs. 2016;22(3):368–91. doi: 10.1177/1074840716656815 27385260

[10] Coleman CI, Coleman SM, Vanderpoel J, Nelson W, Colby JA, Scholle JM, et al. Factors associated with “caregiver burden” for atrial fibrillation patients. Int J Clin Pract. 2012 Oct;66(10):984–90. doi: 10.1111/j.1742-1241.2012.02996.x 22994332

[11] Schron EB, Jenkins LS. Quality of life in older patients with atrial fibrillation. Am J Geriatr Cardiol. 2005 Apr;14(2):87–90. doi: 10.1111/j.1076-7460.2005.02283.x 15785150

[12] Taylor EC, O’Neill M, Hughes LD, Carroll S, Moss-Morris R. ‘It’s like a frog leaping about in your chest’: Illness and treatment perceptions in persistent atrial fibrillation. Br J Health Psychol. 2018 Feb;23(1):3–21. doi: 10.1111/bjhp.12267 28875586

[13] Rosenstrøm S, Risom SS, Ejlertsen C, Hove JD, Brødsgaard A. Dancing with atrial fibrillation–How arrhythmia affects everyday life of family members: A qualitative study. Brunner-La Rocca HP, editor. PLOS ONE 2021 Jul 6;16(7):e0254130. doi: 10.1371/journal.pone.0254130 34228743 PMC8259977

[14] Rosenstrøm S, Risom SS, Hove JD, Brødsgaard A. Living with Atrial Fibrillation: A Family Perspective. Grypdonck MHF, editor. Nurs Res Pract. 2022 Mar 4;2022:1–10. doi: 10.1155/2022/7394445 35280493 PMC8916854

[15] Kirchhof P, Benussi S, Kotecha D, Ahlsson A, Atar D, Casadei B, et al. 2016 ESC Guidelines for the management of atrial fibrillation developed in collaboration with EACTS. Eur Heart J. 2016 Oct 7;37(38):2893–962. 27567408 10.1093/eurheartj/ehw210

[16] Årestedt L, Persson C, Benzein E. Living as a family in the midst of chronic illness. Scand J Caring Sci. 2014 Mar;28(1):29–37. doi: 10.1111/scs.12023 23317153

[17] Ostlund U, Persson C. Examining Family Responses to Family Systems Nursing Interventions: An Integrative Review. J Fam Nurs. 2014 Aug;20(3):259–86. doi: 10.1177/1074840714542962 25026964

[18] Srisuk N, Cameron J, Ski CF, Thompson DR. Heart failure family-based education: a systematic review. Patient Educ Couns. 2016 Mar;99(3):326–38. doi: 10.1016/j.pec.2015.10.009 26519992

[19] Hendriks JML, de Wit R, Crijns HJGM, Vrijhoef HJM, Prins MH, Pisters R, et al. Nurse-led care vs. usual care for patients with atrial fibrillation: results of a randomized trial of integrated chronic care vs. routine clinical care in ambulatory patients with atrial fibrillation. Eur Heart J. 2012 Nov;33(21):2692–9. doi: 10.1093/eurheartj/ehs071 22453654

[20] Gallagher C, Elliott AD, Wong CX, Rangnekar G, Middeldorp ME, Mahajan R, et al. Integrated care in atrial fibrillation: a systematic review and meta-analysis. Heart. 2017 May 10;heartjnl-2016-310952. doi: 10.1136/heartjnl-2016-310952 28490616

[21] Palm P, Qvist I, Rasmussen TB, Christensen SW, Håkonsen SJ, Risom SS. Educational interventions to improve outcomes in patients with atrial fibrillation—a systematic review. Int J Clin Pract [Internet]. 2020 Nov [cited 2021 Nov 30];74(11). Available from: https://onlinelibrary.wiley.com/doi/10.1111/ijcp.13629 32726511 10.1111/ijcp.13629

[22] Østergaard B, Mahrer-Imhof R, Wagner L, Barington T, Videbæk L, Lauridsen J. Effect of family nursing therapeutic conversations on health-related quality of life, self-care and depression among outpatients with heart failure: A randomized multi-centre trial. Patient Educ Couns. 2018;101(8):1385–93. doi: 10.1016/j.pec.2018.03.006 29567335

[23] Voltelen B, Konradsen H, Østergaard B. Family Nursing Therapeutic Conversations in Heart Failure Outpatient Clinics in Denmark: Nurses’ Experiences. J Fam Nurs. 2016 May;22(2):172–98. doi: 10.1177/1074840716643879 27165753

[24] Benzein EG, Saveman BI. Health-promoting conversations about hope and suffering with couples in palliative care. Int J Palliat Nurs. 2008 Sep;14(9):439–45. doi: 10.12968/ijpn.2008.14.9.31124 19060795

[25] Grant S, Mayo-Wilson E, Montgomery P, Macdonald G, Michie S, Hopewell S, et al. CONSORT-SPI 2018 Explanation and Elaboration: guidance for reporting social and psychological intervention trials. Trials. 2018 Dec;19(1):406. doi: 10.1186/s13063-018-2735-z 30060763 PMC6066913

[26] Shajani, Zahra, Snell, Diana. Nurses and Families. A guide to Family Assessment and Intervention. Vol. 2019. F.A.DAVIS.

[27] Harris PA, Taylor R, Thielke R, Payne J, Gonzalez N, Conde JG. Research electronic data capture (REDCap)—A metadata-driven methodology and workflow process for providing translational research informatics support. J Biomed Inform. 2009 Apr;42(2):377–81. doi: 10.1016/j.jbi.2008.08.010 18929686 PMC2700030

[28] Crane S, Comer RS, Arenson AD, Draucker C. Using REDCap to Facilitate Web-Based Therapeutic Intervention Research: Nurs Res. 2019;68(6):483–7. doi: 10.1097/NNR.0000000000000367 31693554 PMC6883142

[29] Akobeng AK. Assessing the Validity of Clinical Trials. J Pediatr Gastroenterol Nutr. 2008 Sep;47(3):277–82. doi: 10.1097/MPG.0b013e31816c749f 18728521

[30] Bell JM. Family Systems Nursing: re-examined. J Fam Nurs. 2009 May;15(2):123–9. doi: 10.1177/1074840709335533 19423766

[31] Bell JM, Wright LM. The Illness Beliefs Model: advancing practice knowledge about illness beliefs, family healing, and family interventions. J Fam Nurs. 2015 May;21(2):179–85. doi: 10.1177/1074840715586889 25995203

[32] Wright Leahey. Nurses and Families- A Guide to Family Assesment and Intervention. 6 udg. Philadelpia: F.A. Davis Company; 2013. 349 p.

[33] Duhamel F, Dupuis F, Wright L. Families’ and Nurses’ Responses to the “One Question Question”: Reflections for Clinical Practice, Education, and Research in Family Nursing. J Fam Nurs. 2009 Nov;15(4):461–85. doi: 10.1177/1074840709350606 19858280

[34] Spertus J, Dorian P, Bubien R, Lewis S, Godejohn D, Reynolds MR, et al. Development and validation of the Atrial Fibrillation Effect on QualiTy-of-Life (AFEQT) Questionnaire in patients with atrial fibrillation. Circ Arrhythm Electrophysiol. 2011 Feb;4(1):15–25. doi: 10.1161/CIRCEP.110.958033 21160035

[35] Holmes DN, Piccini JP, Allen LA, Fonarow GC, Gersh BJ, Kowey PR, et al. Defining Clinically Important Difference in the Atrial Fibrillation Effect on Quality-of-Life Score: Results From the Outcomes Registry for Better Informed Treatment of Atrial Fibrillation. Circ Cardiovasc Qual Outcomes [Internet]. 2019 May [cited 2021 Sep 29];12(5). Available from: https://www.ahajournals.org/doi/10.1161/CIRCOUTCOMES.118.005358 10.1161/CIRCOUTCOMES.118.005358 31092022

[36] Bjelland I, Dahl AA, Haug TT, Neckelmann D. The validity of the Hospital Anxiety and Depression Scale. J Psychosom Res. 2002 Feb;52(2):69–77. 11832252 10.1016/s0022-3999(01)00296-3

[37] Stern AF. The Hospital Anxiety and Depression Scale. Occup Med. 2014 Jul 1;64(5):393–4. doi: 10.1093/occmed/kqu024 25005549

[38] Christensen AV, Dixon JK, Juel K, Ekholm O, Rasmussen TB, Borregaard B, et al. Psychometric properties of the Danish Hospital Anxiety and Depression Scale in patients with cardiac disease: results from the DenHeart survey. Health Qual Life Outcomes. 2020 Dec;18(1):9. doi: 10.1186/s12955-019-1264-0 31910859 PMC6947856

[39] Lemay KR, Tulloch HE, Pipe AL, Reed JL. Establishing the Minimal Clinically Important Difference for the Hospital Anxiety and Depression Scale in Patients With Cardiovascular Disease. J Cardiopulm Rehabil Prev. 2019 Nov;39(6):E6–11. doi: 10.1097/HCR.0000000000000379 30489438

[40] Wynn GJ, Todd DM, Webber M, Bonnett L, McShane J, Kirchhof P, et al. The European Heart Rhythm Association symptom classification for atrial fibrillation: validation and improvement through a simple modification. Eur Eur Pacing Arrhythm Card Electrophysiol J Work Groups Card Pacing Arrhythm Card Cell Electrophysiol Eur Soc Cardiol. 2014 Jul;16(7):965–72. doi: 10.1093/europace/eut395 24534264 PMC4070972

[41] Sveinbjarnardottir EK, Svavarsdottir EK, Hrafnkelsson B. Psychometric Development of the Iceland-Expressive Family Functioning Questionnaire (ICE-EFFQ). J Fam Nurs. 2012 Aug;18(3):353–77. doi: 10.1177/1074840712449204 22752795

[42] Konradsen H, Dieperink KB, Lauridsen J, Sorknaes AD, Ostergaard B. Validity and reliability of the Danish version of the Ice Expressive Family Functioning and Ice Family Perceived Support questionnaires. Scand J Caring Sci. 2018 Dec;32(4):1447–57. doi: 10.1111/scs.12591 30011066

[43] Sveinbjarnardottir EK, Svavarsdottir EK, Hrafnkelsson B. Psychometric Development of the Iceland-Family Perceived Support Questionnaire (ICE-FPSQ). J Fam Nurs. 2012 Aug;18(3):328–52. doi: 10.1177/1074840712449203 22821443

[44] Dorian P, Burk C, Mullin CM, Bubien R, Godejohn D, Reynolds MR, et al. Interpreting changes in quality of life in atrial fibrillation: How much change is meaningful? Am Heart J. 2013 Aug;166(2):381–387.e8. doi: 10.1016/j.ahj.2013.04.015 23895823

[45] R Core Team (2019). R: A language and environment for statistical computing. R Foundation for Statistical Computing, Vienna, Austria. URL https://www.R-project.org/.

[46] WMA—The World Medical Association-WMA Declaration of Helsinki–Ethical Principles for Medical Research Involving Human Subjects [Internet]. [cited 2018 Mar 14]. Available from: https://www.wma.net/policies-post/wma-declaration-of-helsinki-ethical-principles-for-medical-research-involving-human-subjects/

[47] Voltelen B, Konradsen H, Østergaard B. Ethical considerations when conducting joint interviews with close relatives or family: an integrative review. Scand J Caring Sci. 2018 Jun;32(2):515–26. doi: 10.1111/scs.12535 28994460

[48] Recommendations for the protection of rehttp://www.icmje.org/recommendations/browse/roles-and-responsibilities/protection-of-research-participants.htmlsearch Participants.

[49] WHO | The Declaration of Helsinki and public health [Internet]. WHO. [cited 2018 Mar 14]. Available from: http://www.who.int/bulletin/volumes/86/8/08-050955/en/

[50] Wändell P, Carlsson AC, Gasevic D, Wahlström L, Sundquist J, Sundquist K. Depression or anxiety and all-cause mortality in adults with atrial fibrillation–A cohort study in Swedish primary care. Ann Med. 2016 Jan 8;48(1–2):59–66. doi: 10.3109/07853890.2015.1132842 26758363 PMC4790080

[51] Gisi B, Althouse AD, Mathier AS, Pusateri A, Rollman BL, LaRosa A, et al. The unmeasured burden: Contribution of depression and psychological stress to patient-reported outcomes in atrial fibrillation. Int J Cardiol. 2020 Mar;302:75–80. doi: 10.1016/j.ijcard.2019.12.004 31837900 PMC7002250

[52] Moore GF, Evans RE, Hawkins J, Littlecott H, Melendez-Torres GJ, Bonell C, et al. From complex social interventions to interventions in complex social systems: Future directions and unresolved questions for intervention development and evaluation. Evaluation. 2019 Jan;25(1):23–45. doi: 10.1177/1356389018803219 30705608 PMC6330692

[53] Craig P, Dieppe P, Macintyre S, Michie S, Nazareth I, Petticrew M. Developing and evaluating complex interventions: the new Medical Research Council guidance. BMJ. 2008 Sep 29;a1655. doi: 10.1136/bmj.a1655 18824488 PMC2769032

[54] Joensen A, Dinesen P, Svendsen L, Hoejbjerg T, Fjerbaek A, Andreasen J, et al. Effect of patient education and physical training on quality of life and physical exercise capacity in patients with paroxysmal or persistent atrial fibrillation: A randomized study. J Rehabil Med. 2019;0. doi: 10.2340/16501977-2551 30931484

[55] Mark DB, Anstrom KJ, Sheng S, Piccini JP, Baloch KN, Monahan KH, et al. Effect of Catheter Ablation vs Medical Therapy on Quality of Life Among Patients With Atrial Fibrillation: The CABANA Randomized Clinical Trial. JAMA. 2019 Apr 2;321(13):1275. doi: 10.1001/jama.2019.0692 30874716 PMC6450275

[56] Bandura A. Health Promotion by Social Cognitive Means. Health Educ Behav. 2004 Apr;31(2):143–64. doi: 10.1177/1090198104263660 15090118

[57] Petursdottir AB, Svavarsdottir EK. The effectivness of a strengths‐oriented therapeutic conversation intervention on perceived support, well‐being and burden among family caregivers in palliative home‐care. J Adv Nurs. 2019 Nov;75(11):3018–31. doi: 10.1111/jan.14089 31162698

