# Supplementary material for: Clinical outcomes of a short-term family-focused intervention for patients with atrial fibrillation–A randomised clinical trial
Source: PLoS One. 2023 Mar 16;18(3):e0282639. doi: 10.1371/journal.pone.0282639 (PMC10019651; doi:10.1371/journal.pone.0282639)
Supplement: S1 File — (DOCX) [file pone.0282639.s002.docx]

| **Variable** | **Change**  **Difference (control - intervention)** | **p-value*** |
| --- | --- | --- |
| **AFEQT** |  |  |
| AFEQT global score | -4.21 (-9.33 – 9.01) | 0.910 |
| Symptoms | 1.04 (-5.04 – 13.87) | 0.356 |
| Daily activities | -3.26 (-14.40 – 18.01) | 0.709 |
| Treatment concern | -2.22 (-6.96 – 0.61) | 0.161 |
| Treatment satisfaction | -3.33 (-24.12 – 5.83) | 0.370 |
| **HADS** |  |  |
| Anxiety | -0.50 (-1.91 – 0.93) | 0.582 |
| Depression | -0.25 (-3.22 – 0.18) | 0.434 |
| **EHRA score** | 0.00 (0.00 – 0.55) | 0.929 |
| **ICE-EFFQ** |  |  |
| Expressive emotions | 0.25 (-0.70 – 1.19) | 0.420 |
| Collaboration  /problem solving | 0.46 (-1.01 – 1.95) | 0.870 |
| Communication | -0.86 (-1.80 – -0.021) | 0.149 |
| Behaviour | -0.29 (-3.41 – 1.03) | 0.165 |
| **Total ICE-EFFQ** | -0.33 (-5.12 – 2.89) | 0.351 |
| **ICE-FPSQ** |  |  |
| Cognitive support | -8.80 (-10.46 – -4.76) | <0.001 |
| Emotional support | -28.87 (-30.88 – -21.09) | <0.001 |
| **Total ICE-FPSQ** | -35.41 (-40.81 – -26.01) | <0.001 |
