# Supplementary material for: Clinical outcomes of a short-term family-focused intervention for patients with atrial fibrillation–A randomised clinical trial
Source: PLoS One. 2023 Mar 16;18(3):e0282639. doi: 10.1371/journal.pone.0282639 (PMC10019651; doi:10.1371/journal.pone.0282639)
Supplement: S2 File — (PDF) [file pone.0282639.s003.pdf]

Protocol PhD study / November 2018

A Family focused Nursing Intervention for Patients with Atrial Fibrillation – Effect of Group Education and Family Strength Orientated Therapeutic Conversations

Project group

PhD student/Project manager:

Stine Maria Rosenstrøm

RN, Master of Health Science

Department of Cardiology -253

Kettegård Allé 30

DK- 2650 Hvidovre

Email: stine.maria.rosenstroem@regionh.dk/ Phone: +4530277208

Main supervisor:

Anne Brødsgaard

Senior researcher & associate professor, RN, MPH, PhD, at Amager Hvidovre Hospital & Aarhus University

Departments of Pediatrics-460

Kettegård Allé 30

DK-2650 Hvidovre

Email: anne.broedsgaard.madsen@regionh.dk Phone work: +45 38623129

Project supervisors:

Signe Stelling Risom

RN, MHSc, PhD,

The Heart Centre, Unit 2151, Rigshospitalet, Inge Lehmanns Vej 7, 2100 Copenhagen O, University College Copenhagen, Tagensvej 86, 2200 Copenhagen N

Email: signe.stelling.risom@regionh.dk / Phone: +45 51632786

Jens Dahlgaard Hove

MD, MSc, PhD, Associate Professor at Amager Hvidovre Hospital & University of Copenhagen.

Department of Cardiology -253

Kettegård Allé 30

DK- 2650 Hvidovre

Email: jens.dahlgaard.Hove.@regionh.dk/ Phone: +45 20235445

Reference group/ members of the project group:

Dorrit Hjelt Thorsen, RN, MHM, Head Nurse, Department of Medicine-147, Kettegård Allé 30, 2650 Hvidovre.

Vibeke Nørholm, RN, MSc, PhD, Senior researcher at Clinical Research Centre Kettegård Allé 30, 2650 Hvidovre.

Sussie Foghmar, RN, Department nurse, Department of Cardiology-253, Kettegård Allé 30, 2650 Hvidovre.

Ulrik Dixen, MD, PhD, associate professor, Amager & Hvidovre Hospital & University of Copenhagen. Department of Cardiology -253, Kettegård Allé 30

Thomas Kallemose, MSc in statistics, at Clinical Research Centre Kettegård Allé 30, 2650 Hvidovre.

Locality of the study:

Amager & Hvidovre Hospital, Department of Cardiology-253, Kettegård Allé 30, 2650 Hvidovre. Denmark

Background

Atrial fibrillation (AF) is the most common cardiac arrhythmia and is often described as the new epidemic among heart diseases (1). In 2010 the estimated worldwide numbers of men and women with AF were 20.9 million and 12.6 million, respectively, with higher incidence and prevalence rates in the developed countries (2). In Denmark, the prevalence of AF is approximately 93,000 and the incidence about 20,000 new cases per year (3). Estimated number AF-patient in local department is 400/ year.

AF is a costly public health problem with hospitalizations as the primary expenditure (4). The disease and treatment is complex and requires a high degree of adherence to prevent complications (stroke and major bleedings) and the burden of symptoms often leads to poor health related quality of life (HRQOL). The impairment of HRQOL due to AF can be comparable to that observed in patients with heart failure which is due to both the often disabling symptoms of the disease, and the risk of complications such as stroke (5)(6). The consequences of AF are often impairment of physical functioning and mental health (7). A qualitative study by McCabe et al. found that AF patients often are anxious. They have feelings of nonsupport, inadequate self-management counseling, and distress associated with the unpredictable symptoms (8).

Illness is considered to be a family affair and it is therefore important for patients and their families to have a chance to talk about issues like suffering and coping (9). Families need support and solutions to ensure successful adjustments to adequately cope with a family member with chronic illness (10). Little is known about how families experience to habituate life with AF, their concerns and feelings and their need for support. One study found that the greatest burden to family members of AF patients occur due to disruption in schedule, followed by financial problems, lack of family support and health problems (11). It has also been described how couples where one suffer from AF often experience uncertainty as a common main concern which is rooted in not knowing enough about the disease (12). Studies have shown that health care providers should not only focus on the patient with AF but should take the family into account as well, when managing this condition because the feelings and illness beliefs affect the entire family (13). Future research and intervention should therefore include the whole family.

Hendriks et al. studied the effect of nurse-led care (focused on AF guidelines and patient information) versus usual care for patients with AF. Nurse-led care was superior to usual care in terms of cardiovascular hospitalizations and cardiovascular mortality (14). These findings have generated more attention towards patients with AF and the possibility that they could benefit from specific disease management and rehabilitation similar to programmes used in other chronic cardiovascular conditions such as heart failure and ischemic heart disease (15)(16). However, no clinical guidelines ensure patients and families education to cope with AF. Furthermore, the limited intervention studies are tested in different settings with AF-patients and with different clinical intervention contents. European Society of Cardiology recommend that more research about care for patients with AF are needed and it is likely to require different designs in different healthcare settings (2). More research should focus on testing new intervention models that could benefit patients and their families.

Family focused Nursing (FFN) with reference to the The Calgary Family Assessment model (CFAM) and the Calgary Family Intervention Model (CIFM) used as a theoretical model have been shown to be helpful in nursing interventions supporting patient and family in managing life with diabetes (17). However the efficacy of these models have never been tested in nursing interventions

involving patients with AF and the possible potential still needs to be proved (12) (18). Optimization of quality and coherence of the patient experience are recommended for patients with heart diseases, where patients and families are the center point of care (16). Unfortunately, these visions are not always realized in practice for patients with AF (19). Therefore, more research is needed about how healthcare professionals can involve, support and educate AF-patients and their families. In future studies of FFN a new pilot study about how AF-patients experience the patient course should be taking in to account. The themes were *"Uncertainty as a companion in the process", "To understand what is wrong with me", and "to put the heart at the right place"*(20).

The rising number of patients with AF, the complexity of the disease and treatment, the frequent non-adherence to treatment, the poor QoL, and lack of support for the family demands attention in the management of AF and in how patients and their families cope with the disease. Evidence of the effect of integrating the family in the counselling of patients are in general sparse (21). To facilitate evidence-based knowledge this study is designed to test a health promoting intervention for patients with AF and their families.

Aim and research questions

The aim of the study is to examine how AF- patients and families are influenced by AF and to test if involving family members in a FFN intervention with group education improves outcomes for patients with AF. The aim can be achieved by answering the following research questions.

Study 1: How does family members and AF patients experience family life being affected by AF?

Study 2: How do family members experience life with a family member having AF?

Study 3: Is a short-term educational and family focused support intervention for patients and family members living with AF more effective on quality of life than standard care?

Method

The study will consist of three sub-studies. A qualitative explorative study with family unit interviews, focus group interview with members of the family, and a quantitative fidelity study evaluating the effect of a family focused nursing intervention based on CFAM and CIFM

Materials

Inclusion criteria: Men and women > 18 years with newly diagnosed AF (< 4 weeks since diagnose) documented in the patient file and verified by a EKG. Family members must be > 18 years old and defined by the patient: e.g. spouse, son, daughter, near friend or neighbor.

Exclusion criteria: Patients with chronic heart failure according to international guidelines (2).

Study part 1: Family unit interviews

Qualitative data with patients and family members will be collected from a semi-structured interview-guide founded in a structured literature search. Families will be recruited using a purposeful sampling strategy. This method is used to find participants who collectively provide a

rich and varied data material. (e.g. difference in gender, age, income, and symptoms). The family interviews will continue until data saturation has been achieved (22) which is estimated to be approximately 15-20 patients including family members. Families will be interviewed at home or at the hospital according to their preferences. A qualitative methodological approach employing Giorgi's descriptive phenomenological method will be used to collect and analyse data which follows four steps 1: The data is read to give an overall impression. 2: Meaningful units are identified. 3: Meaningful units are transformed into categories. 4: Categories are synthesised into the essence of the phenomenon. The researcher's disciplinary perspective and professional sensitivity will guide the process (23) and the researchers preunderstanding will be revealed by a peer interview prior to the data collection. The study will broaden the perspective of how the family are influenced by AF, their thoughts and feelings can be used as inspiration in the intervention study and benefit in how to support AF patients in clinical health care.

Study Two: Focus group interviews with family members

Data will be collected from 2 - 3 focus group interviews each with approximately 5-8 family members. Focus group interviews can be helpful when facilitating the perspectives and opinions of the family members (24). The motive is to attain a condensed and broad description of a phenomenon (25) (26) and to bring positive dynamics into the focus groups, which can help informants to talk about feelings regarding their experiences of being part of a family where a family member has AF. Interview data will be recorded and transcribed ad verbatim and will be analyzed with qualitative content analysis (21). Content analysis is a research method widely used in health care research and it is known for making replicable and valid conclusions from data to their context, with the purpose of providing knowledge, new insights, a representation of facts and a practical guide to action (26).

The gathered knowledge from study 1 and 2 will be used to test the fidelity of a health promoting intervention for AF patients and their families.

Study three: Fidelity study

The fidelity study is designed as a randomized clinical trial. Based on sample-size calculation 100 patients will be allocated in two groups by a computer program. An intervention group (n=50), and a control group (n=50), respectively. The intervention group will receive health promoting group education and sessions based on the theoretical framework of FFN including CFAM and CFIM as a supplement to conventional care and treatment according to international guidelines (2). The interventional focus is on the psychological and educational needs of patients with AF and their families (e. g medication, handling of symptoms, managing stress and anxiety, facilitating the lifestyle changes, exercising, returning to work, and taking part in family and social activities)(27).

The control group will receive conventional care and treatment according to guidelines (2). Conventional care is characterized by ad hoc management as per usual standards of clinical care in Denmark (with access to routine medical care, hospital care, and pharmacotherapy). Patients will be assigned to the fidelity study according to the described in- and exclusion criteria.

The intervention will take place in the Department of Cardiology at Amager Hvidovre Hospital (AAH) and the inclusion period is estimated to one year.

Description of the intervention:

In FFN based on CFAM and CIFM the whole family is considered the unit of assessment and intervention when a member of the family becomes ill (28). In FFN the focus is therefore not only on the patient but on the interaction and the reciprocity between family members, patient and between the nurse. FFN is operationalized within a therapeutic relationship and conversations between a patient / family and a nurse. The idea is to soften illness suffering by creating a context for changing the perspective on illness beliefs and facilitating beliefs that can strengthen the psychological resilience (29). To guide the patient and the families the Family Strength Orientated Therapeutic Conversations (FAM-SOTC) will be used as health promoting conversations and a way to enhance family health (28). The therapeutic conversations build on different theories: System theory, change theory and communication theory. Focusing on illness narrative, therapeutic questions, strengths, identification on psychological endurance and resources and through evidence-based information and recommendation (10).

First part of the intervention is a two hour group session designed by the PhD student and the project nurses based on clinical guidelines of AF-management (2) and theory from multifamily group intervention (30). Nurse specialists will facilitate knowledge to patients and family members about AF and how to support self-management in their daily living. Furthermore during six months´ patients in the intervention group will receive 3-5 FAMT SOCT sessions of 30-60 minutes. First session will start as soon as possible and the nurse, patient and family will plan the numbers and session intervals according to the patient and the family needs of support and guidance. The PhD student and two project nurses from the ward will be trained theoretically and practically in performing health promoting family conversations at Linné University in Sweden.

Study design, study outcomes, and statistical methods

To assess the fidelity of the study, a process evaluation will be carried out (31). This will be done by recording all nursing sessions. It will be used to evaluate if the planned intervention is carried out as intended. Process evaluation is very suitable to evaluate the process and to give an estimate of the fidelity of the intervention. It can also explain reasons for success and failure in outcome results (31).

The influence of the intervention is measured quantitatively using internationally validated questionnaires. Measurements of the effect will be collected 24-48 hours after enrollment and follow-up will be made by telephone interviews after 6 months. RedCap database will be used for collecting data and storing data from questionnaires.

The primary outcome is Quality of Life living with AF. This will be accessed with the Atrial Fibrillation Effect on QualiTY-of-Life (AFEQT) Questionnaire (32). Secondary outcomes of interest are family function which will be measured with the Ice Expressive Family Functioning Questionnaire (ICE-EFFQ) and the Ice Family Perceived Support (ICE-FPSQ) (33). Anxiety scores will be measured with the Hospital Anxiety Depression Scale (HADS) (34), AF symptoms measured with the EHRA scale (European Heart Rhythm Association) (35) and number of hospital admissions registered from The National Patient Registry.

Baseline data will be compared between the intervention group and the standard care group. For demographic data Pearsons X^{2-} test or Fisher´s exact test will be used. Depending on the distribution the independent t-test or Wilcoxon rank-sum test will be used. Normally distributed data will be presented with a mean and a standard deviation (SD), non-parametric data with median and inter quartile range. It is estimated that a sample size of 50 patients in two arms (100 patients total) will

make it possible to detect a difference of 11 points on the AFEQT (36) with a power of 80 % and a significance level of 0.05. Calculation are based on a two-sided independent two-sample t-test. An increase between 6 and 19 points has been suggested to correspond to a meaningful improvement in Quality of life measured on AFEQT (36).

Ethical consideration and approvals

The study will be registered to the Danish Data Protection Agency under The Capital Region and the Scientific Ethical Committee. All participants in the studies will receive written and oral information about the study and provide written informed consent regarding participation, which they can retract at any time in accordance with the Helsinki declaration (37). All data will be treated confidentially and anonymized. Qualitative data are stored securely in locked electronic file at AHH and data from quantitative data are securely stored in RedCap database.

Presentations

Three scientific manuscripts will be written with the following preliminary titles for publishing in international journals:

- Family function and atrial fibrillation- from a perspective of the patient and family.
- Living with atrial fibrillation- experiences from family members.
- A prospective randomized controlled fidelity study of an educational and family focused intervention for patients with atrial fibrillation

Negative, inconclusive and/or positive results will be published and communicated nationally and internationally in peer-reviewed health journals and presented at symposias and interdisciplinary conferences.

Perspectives

It is expected that the study will provide a new understanding and evidence based knowledge on how to incorporate health promoting conversations and education of patients and their families into the AF management and care. In the future, results from the fidelity study can be used to design a randomized controlled trial which could determine the possibilities and limitations of a tailored health promotion intervention for patients with AF, expecting to benefit both patients with AF and their families.

The study plan and financing

The project is expected to be initiated in February 2019 and it will be completed in three years (appendix 1.) In 2019, the first two sub-studies will be performed and education of project nurses will take place. In 2020, the third sub-study with intervention will be performed, and in 2021 all data and results will be analyzed and published. Ongoing financial support is being sought from relevant funding sources. Guarantee for cover of all payroll costs has been given by the Department of Medicine (Medicinsk Enhed) at AAH in case external funding is not obtained.

Litteraturliste

1. Chugh SS, Blackshear JL, Shen WK, Hammill SC, Gersh BJ. Epidemiology and natural history of atrial fibrillation: clinical implications. J Am Coll Cardiol. februar 2001;37(2):371–8.

2. Kirchhof P, Benussi S, Kotecha D, Ahlsson A, Atar D, Casadei B, m.fl. 2016 ESC Guidelines for the management of atrial fibrillation developed in collaboration with EACTS. Eur Heart J. 7. oktober 2016;37(38):2893–962.

3. hjertekarsygdomme_i_2011-2_rapport.pdf [Internet]. [henvist 30. marts 2017]. Tilgængelig hos: http://www.si-folkesundhed.dk/upload/hjertekarsygdomme_i_2011-2_rapport.pdf

4. Ringborg A, Nieuwlaat R, Lindgren P, Jönsson B, Fidan D, Maggioni AP, m.fl. Costs of atrial fibrillation in five European countries: results from the Euro Heart Survey on atrial fibrillation. Eur Eur Pacing Arrhythm Card Electrophysiol J Work Groups Card Pacing Arrhythm Card Cell Electrophysiol Eur Soc Cardiol. april 2008;10(4):403–11.

5. Jenkins LS, Brodsky M, Schron E, Chung M, Rocco T, Lader E, m.fl. Quality of life in atrial fibrillation: the Atrial Fibrillation Follow-up Investigation of Rhythm Management (AFFIRM) study. Am Heart J. januar 2005;149(1):112–20.

6. Schron EB, Jenkins LS. Quality of life in older patients with atrial fibrillation. Am J Geriatr Cardiol. april 2005;14(2):87–90.

7. Altiok M, Yilmaz M, Rencüsoğullari I. Living with Atrial Fibrillation: An Analysis of Patients' Perspectives. Asian Nurs Res. december 2015;9(4):305–11.

8. McCabe PJ, Schumacher K, Barnason SA. Living with atrial fibrillation: a qualitative study. J Cardiovasc Nurs. august 2011;26(4):336–44.

9. Benzein EG, Saveman B-I. Health-promoting conversations about hope and suffering with couples in palliative care. Int J Palliat Nurs. september 2008;14(9):439–45.

10. Lorraine M. Wright, Leahey M. Nurses and Families A Guide to Family Assesment. 6. udg. Philidelphia: F.A. Davis Company; 2013.

11. Coleman CI, Coleman SM, Vanderpoel J, Nelson W, Colby JA, Scholle JM, m.fl. Factors associated with “caregiver burden” for atrial fibrillation patients. Int J Clin Pract. oktober 2012;66(10):984–90.

12. Dalteg T, Benzein E, Sandgren A, Fridlund B, Malm D. Managing uncertainty in couples living with atrial fibrillation. J Cardiovasc Nurs. juni 2014;29(3):E1-10.

13. Dalteg T, Benzein E, Sandgren A, Malm D, Årestedt K. Associations of Emotional

Distress and Perceived Health in Persons With Atrial Fibrillation and Their Partners Using the Actor-Partner Interdependence Model. J Fam Nurs. 2016;22(3):368–91.

14. Hendriks JML, de Wit R, Crijns HJGM, Vrijhoef HJM, Prins MH, Pisters R, m.fl. Nurse-led care vs. usual care for patients with atrial fibrillation: results of a randomized trial of integrated chronic care vs. routine clinical care in ambulatory patients with atrial fibrillation. Eur Heart J. november 2012;33(21):2692–9.

15. Risom SS, Zwisler A-D, Johansen PP, Sibilitz KL, Lindschou J, Gluud C, m.fl. Exercise-based cardiac rehabilitation for adults with atrial fibrillation. Cochrane Database Syst Rev [Internet]. 2017 [henvist 13. november 2018];(2). Tilgængelig hos: https://www.cochranelibrary.com/cdsr/doi/10.1002/14651858.CD011197.pub2/abstract

16. Hendriks J, Tomini F, van Asselt T, Crijns H, Vrijhoef H. Cost-effectiveness of a specialized atrial fibrillation clinic vs. usual care in patients with atrial fibrillation. Eur Eur Pacing Arrhythm Card Electrophysiol J Work Groups Card Pacing Arrhythm Card Cell Electrophysiol Eur Soc Cardiol. august 2013;15(8):1128–35.

17. Eva Benzein, Hagberg M. Familjefokuserad omvårdnad - ett strategidokument. Linnéuniversitet Institutionen for hälso - og vårdvetenskab 82 Kalmar 391e.v.t.;Centrum for forskning om familiers hälsa 2010.

18. The heart is a representation of life: an exploration of illness beliefs in couples living with atrial fibrillation - Dalteg - 2017 - Journal of Clinical Nursing - Wiley Online Library [Internet]. [henvist 8. oktober 2018]. Tilgængelig hos: https://onlinelibrary-wiley-com.ep.fjernadgang.kb.dk/doi/pdf/10.1111/jocn.13742

19. hjertekarpatienters_oplevelser_med_sundhedsvæsenet_og_livet_med_en_hjertekarsygdom.pdf [Internet]. [henvist 23. juni 2017]. Tilgængelig hos: http://www.si-folkesundhed.dk/upload/hjertekarpatienters_oplevelser_med_sundhedsv%C3%A6senet_og_livet_med_en_hjertekarsygdom.pdf

20. Rosenstrøm, Stine. Patients with atrial fibrillation and their experiences and perspectives on the patient course. Københavns Universitet; 2018.

21. Ostlund U, Persson C. Examining Family Responses to Family Systems Nursing Interventions: An Integrative Review. J Fam Nurs. august 2014;20(3):259–86.

22. Kvale Steinar, Brinkmann Svend. Interview, introduktion til et håndværk. 2. udgave, Steinar Kvale, Svend Brinkmann, 1. oplag. Kbh: Hans Reitzel; 2009.

23. Giorgi A. The Phenomenological Movement and Research in the Human Sciences. Nurs Sci Q. 1. januar 2005;18(1):75–82.

24. Halkier B. Fokusgrupper. Bd. 2016. Samfundslitteratur; 126 s.

25. Elo S, Kyngäs H. The qualitative content analysis process. J Adv Nurs. april 2008;62(1):107–15.

26. Graneheim UH, Lundman B. Qualitative content analysis in nursing research: concepts, procedures and measures to achieve trustworthiness. Nurse Educ Today. februar 2004;24(2):105–12.

27. McCabe PJ, Barnason SA. Illness perceptions, coping strategies, and symptoms contribute to psychological distress in patients with recurrent symptomatic atrial fibrillation. J Cardiovasc Nurs. oktober 2012;27(5):431–44.

28. Benzein EG, Hagberg M, Saveman B-I. "Being appropriately unusual": a challenge for nurses in health-promoting conversations with families. Nurs Inq. juni 2008;15(2):106–15.

29. Bell JM, Wright LM. The Illness Beliefs Model: advancing practice knowledge about illness beliefs, family healing, and family interventions. J Fam Nurs. maj 2015;21(2):179–85.

30. Østergaard B, Konradsen, Hanne H. Familiesygepleje. 1. Udgave. Bd. 2016. København: Munksgaard Danmark;

31. Hulscher MEJL, Laurant MGH, Grol RPTM. Process evaluation on quality improvement interventions. Qual Saf Health Care. februar 2003;12(1):40–6.

32. Spertus J, Dorian P, Bubien R, Lewis S, Godejohn D, Reynolds MR, m.fl. Development and validation of the Atrial Fibrillation Effect on QualiTy-of-Life (AFEQT) Questionnaire in patients with atrial fibrillation. Circ Arrhythm Electrophysiol. februar 2011;4(1):15–25.

33. Konradsen H, Dieperink KB, Lauridsen J, Sorknaes AD, Ostergaard B. Validity and reliability of the Danish version of the Ice Expressive Family Functioning and Ice Family Perceived Support questionnaires. Scand J Caring Sci. 16. juli 2018;

34. Villoria E, Lara L. Assessment of the Hospital Anxiety and Depression Scale for cancer patients. Rev Med Chil. marts 2018;146(3):300–7.

35. Wynn GJ, Todd DM, Webber M, Bonnett L, McShane J, Kirchhof P, m.fl. The European Heart Rhythm Association symptom classification for atrial fibrillation: validation and improvement through a simple modification. Eur Eur Pacing Arrhythm Card Electrophysiol J Work Groups Card Pacing Arrhythm Card Cell Electrophysiol Eur Soc Cardiol. juli 2014;16(7):965–72.

36. Interpreting changes in quality of life in atrial fibrillation: How much change is meaningful?- ClinicalKey [Internet]. [henvist 6. november 2018]. Tilgængelig hos: https://www-clinicalkey-com.ep.fjernadgang.kb.dk/#!/content/playContent/1-s2.0-S0002870313003554?returnurl=null&referrer=null

37. WHO | The Declaration of Helsinki and public health [Internet]. WHO. [henvist 14.

marts 2018]. Tilgængelig hos: http://www.who.int/bulletin/volumes/86/8/08-050955/en/
